# Lectures on Scarlet Fever

**Published:** 1851-11

**Authors:** Caspar Morris

**Affiliations:** Late Lecturer on Practical Medicine in the Philadelphia Medical Institute


### MEDICAL EXAMINER,

AND

### RECORD OF MEDICAL SCIENCE.

NEW SERIES.—NO. LXXXIII.—NOVEMBER, 1851.

---

### ORIGINAL COMMUNICATIONS.

---

*Lectures on Scarlet Fever.* By Caspar Morris, M. D., late Lecturer on Practical Medicine in the Philadelphia Medical Institute.

### Lecture IX.

We now come to the consideration of the treatment of those diseased conditions which are commonly recognized as the *sequelæ* of scarlet fever, though they may perhaps be considered, with greater propriety, a mere continuance of the diseased action caused by the specific poison.

You will remember that I described them as the consequences of the primary disease, and spoke of them as susceptible of division into two classes. Those which appear to be the mere continuation of the local lesions developed during the progress of the disease itself, ulceration of the throat, enlargement of the cervical glands, with infiltration of the cellular tissue of the adjacent parts, abscesses about the neck, and inflammation of the ear, were grouped together as the first class: while the inflammation of the endocardium, or of the kidneys, and the dropsical effusion and rheumatic fever, were arranged as the second, and an attempt was made to exhibit the connection of the dropsical effusion with the interruption of the functions of the emunctories of the skin and kidneys. This division was not adopted without design, and has reference to the treatment appropriate to the several groups.

The ulceration of the throat requires no change in the treatment which has already been suggested as appropriate to the anginose symptoms during the primary stage. The lime water, solution of chloride of soda, sulphate of copper or nitrate of silver, may be injected as detergent and stimulating applications for the cure of the internal affections, should they not subside spontaneously with the decline of the fever: while fomentations are still applied to the external swellings. If suppuration occur, the abscesses should be discharged so soon as the matter approaches near enough to the surface to permit the safe resort to the lancet.

The affections of the ear which complicate scarlet fever in its progress, or are developed as sequelæ, should always receive prompt attention, as they not unfrequently result in the entire destruction of the diseased organ. There may be *otitis* or acute inflammation, or mere *otorrhœa.* The least important cases are those in which the inflammation commences in the meatus auditorius externus. This may occur at any time during the progress of the disease, but most commonly commences about the fourth or fifth day. If the patient be a young child, it will be found more drowsy than usual, and when awake, more fretful; and if old enough to express the seat of distress, it will indicate one or both ears. In some cases, however, there is little or no pain, and the first intimation of any local lesion will be derived from the stain of the cap, or pillow-case, by a glairy, purulent discharge, which excoriates the concha as it flows over it, and occasions a vesicular eruption wherever it touches the skin. Whether the accumulation of this matter in the ear produces an extension of the disease to the tympanum, or the membrana tympani itself takes on the inflammatory action, it not unfrequently happens that even those cases which are attended by little or no suffering in the beginning, become very serious in their progress, and result, finally, in the destruction of the membrana tympani and discharge of the ossicula.

During the first few days, the external meatus should be kept perfectly cleansed by the injection of simple warm water with a little pure castile soap dissolved in it; after which, if the discharge still continue and become fœtid, the weak solution of chloride of soda, or sulphate of copper may be employed.

The injection of the ear should never be entrusted to the mother or nurse. Ignorant of the structure of the parts, they are either restrained from the effectual use of the remedy by apprehension of doing mischief, or employ an undue force and injure the inflamed membrane. Where the ulcerative process has destroyed the membrana tympani, especially, great care is requisite not to throw the fluid so far into the cavity that it shall lodge there and become a source of additional irritation.

Even the cases which originate externally, sometimes result in the entire destruction of the ear; but where the inflammation is extended from the fauces through the eustachian tube to the internal ear, the pain at the time of invasion is much more severe, and the conseqences are much more serious. In the case of infants or young children, this internal affection is first manifested by sudden shrieks, like those of meningitis, accompanied by grinding of the teeth and violent febrile excitement; these symptoms may continue many days before any discharge from the external ear affords positive evidence of the site of the inflammation. These are the cases which result, of necessity, in the destruction of the organ, and lay the foundation for necrosis of the petrous portion of the temporal bone, and occasionally give rise to inflammation of the dura mater and death. It is not at all uncommon for the matter to find its way into the mastoid cells, and to give rise to abscesses behind the ear, which discharge externally; and leave openings, through which pus, contaminated by the dead bone, is discharged during a series of years; making the patient an object of disgust and pity by the excessive fetor. I have known both ears thus destroyed, reducing the child to the condition of a mute.

This extension of the inflammation from the fauces to the ear, may occur at any period; either during the progress of the scarlet fever, or after the primary symptoms have declined. In either case, it demands prompt and energetic treatment; and unless the prostration of the vital forces is so extreme as to render the recovery of the patient hopeless, *leeches* should be applied to the mastoid process, followed by a blister. No apprehension of sloughing or ulceration should be allowed to interfere. The introduction of warm olive oil and laudanum into the ear may be resorted to for the temporary relief of the suffering, simultaneously with these applications; and morphia may be applied afterward to the blistered surface, in doses appropriate to the age of the patient.

*Croup*, when it occurs as a complication during the first week of the disease, should be treated by emetics of alum or sulphate of zinc. Should it be developed during the convalescence, the disease of the larynx will partake of the character of that of the adjacent parts, and if that be one of active inflammation, leeching may be safely resorted to; the quantity of blood drawn being regulated by the degree of reaction which is present. Cases of this kind form the connecting link between the first class of sequelæ and the second; to the consideration of which I shall next invite your attention.

In taking leave of the primary stage of the disease, and the local affections associated with, or immediately dependent upon it, we encounter an entirely new condition of the system, one of active inflammation.

You will remember that the *malignant* form of scarlet fever is less likely to be followed by those sequelæ which I have grouped together as the second class, than the *simple* and *anginose*. Though in the former, the powers of the system may be so far exhausted that it is long in recovering its healthy tone, and the convalescence may be protracted to many weeks, and rendered more tedious by the condition of the throat or ears, there appears to be little or no tendency to the secondary inflammations or dropsical effusions which so frequently follow even the mildest cases of the latter. These sequelæ are always of an inflammatory character, and in severe cases the safety of the patient depends upon the promptitude and vigor with which depletion is practised. They most frequently occur, you will remember, between the 10th and 20th day from the development of the primary symptoms, and are all of them ushered in by loss of appetite, languor, and, in cases of children, fretfulness; and are generally excited, as you will re member, by errors in diet or exposure to vicissitudes of temperature, though they occur without such exciting cause with sufficient frequency to justify the suspicion that they are, in reality, dependent on the original influence of the miasm which gives rise to the disease of which they would then be merely the latter stage, rather than a sequela. They are all accompanied by a dry, heated, condition of the skin; a suspension or great diminution of the secretion from the kidneys; a tense and rapid pulse.

The endocardial inflammation, though not the most common, is the most formidable of these sequelæ, and may be recognized by the extreme rapidity of the pulse, the disposition to sigh, and especially the pain in the region of the heart. I have already indicated the plan of treatment which I think adapted to this case, when I alluded to it in the description of the disease. A decided and immediate impression should be produced by free venesection; a full dose of calomel should be given, followed by oil, or citrate of magnesia; a blister should be applied over the region of the heart; the diet should be restricted to barley or rice water; the most positive quiet should be maintained; and if the febrile reaction be not subdued by these means, and the strength of the patient admit it, leeches or cups should be applied between the lower angle of the scapula of the left side and the vertebræ. Having by these means subdued the active phlogosis, the patient must be kept in a state of perfect tranquillity of mind and body, and on the most rigid diet, till all symptoms of active inflammation are subdued.

The dropsical effusion and the inflammation of the kidneys may be spoken of under one head, since they are at least intimately associated, and demand the same treatment, even if the former do not generally depend on the latter as the proximate cause, which I believe to be the true pathology of such cases. Where the affection is slight and the febrile reaction mild, a dose of calomel followed by a saline cathartic, will often suffice to subdue the inflammation and arrest the progress of the effusion. Should the skin continue dry and husky, the tongue coated, the pulse quick and corded, great advantage may be expected from the use of the solution of citrate of potash or the effervescing draught with sweet spirits of nitre; and these failing to give relief, the nitrate of potash with antimony and small doses of calomel may be resorted to. If the fever be high and constant, bleeding will be not only well born, but absolutely requisite. The blood drawn under these circumstances is always much cupped, and covered with a dense buffy coat, and the relief which follows the abstraction of it is very great; I have known the most urgent symptoms yield immediately. The first indication of improvement will be found in the restoration of the healthy action of the kidneys. Should this not take place very shortly, digitalis should be given.

The best form for exhibiting this remedy with reference to its diuretic effect, is, certainly, the infusion, made according to the prescription of Withering, except that sweet spirits of nitre may be substituted for the Tinct. Cinnamomi, with great advantage. You will find the mode of preparation given in the article on digitalis in the Dispensatory of Wood and Bache. The dose must of course be proportioned to the age of the patient. If there be much fever, and the urine be very scant and high colored with deposit of a brown flocculent matter, there can be no room to doubt the presence of active congestion or inflammation of the kidney, which will be best met by the application of leeches or cups to the loins. I have found the perseverance in nitre and diuretic remedies in these cases, without first removing the local inflammation by blood-letting, to aggravate the sufferings of the patient without producing any increase of the secretion.

But of all the manifestations of disease which mark the progress of scarlet fever, none are so much to be dreaded as the cerebral affections which occur at the period now under consideration. By some authors we are taught that meningitis is suddenly developed and proves fatal, either by giving rise to serous effusion, or terminating in the secretion of pus, or lymph. I have never met with cases in which I had reason to suspect the results last mentioned. The serous effusion is comparatively of frequent occurrence, and is sometimes poured out so rapidly as to produce convulsions and death, before the proper means for relief could be applied. In other instances the effusion takes place more gradually, but with an equally fatal tendency.

Some years since, I was attending a young gentleman of about 15 years of age, who was apparently convalescent from a severe attack of simple scarlet fever. He was an only son, and the object of intense anxiety to his parents. My directions about diet and exposure were carried out with great care; and yet the fever, and suspension of secretion from the skin and kidneys, supervened at the end of the second week. I at once resorted to purgatives and nitrate of potash. On the third day I was summoned to see him lying entirely comatose, with slow pulse, dilated pupils and cool skin. There was no metastasis of the effusion to the brain, for his whole body was œdematous. Recognizing at once the urgency of the case, I tied up his arm and bled him largely, and then put him on the use of small doses of calomel and nitrate of potash, with the infusion of digitalis. At the end of forty-eight hours the kidneys resumed their natural action, and in less than a week the whole train of symptoms had disappeared. These cerebral symptoms depend on the effusion of fluid into the ventricles or at the base of the brain, rather than on any vascular congestion or inflammation of the membranes, and are consequent upon the same causes as those which give rise to the anasarca; and the bleeding acts by curing the condition of the kidneys upon which the whole series of symptoms depends, as well as by withdrawing a portion of the blood, rendered unhealthy by the retention of matters which should have been eliminated by the skin and kidneys.

The next most formidable of the sequelæ is the effusion into the cavity of the pleura, or the parenchyma of the lungs, giving rise to the most distressing orthopnœa. Depending on the same cause as the cerebral cases just noticed, it requires the same treatment which should be pursued with the same degree of energy.

I have never seen a case of dropsical effusion following scarlet fever, which was not materially aggravated by the resort to tonic or stimulating remedies. When the effusion, whether general or local, has been absorbed and discharged through the kidneys, but not till it has entirelydisappeared, it will be proper to resort to the use of some mild tonic; small doses of quinine in the first instance, followed by some of the preparations of iron, with fresh air and a good diet. It must not, however, be forgotten, that the convalescence from these attacks is slow. And that it is even possible that many months may elapse before the patient is restored to a condition of entire health.

The Rheumatic Fever I have always found yield to properly graduated doses of Dover's powder, or morphia and sweet spirits of nitre. Should the pulse be tense and quick, and the suffering prolonged, bleeding would be appropriate in this case also.

Diarrhœa depends upon the irritation produced by the acrid matter swallowed from the throat, and is most likely to occur after very severe anginose or malignant cases. It is quite possible that ulceration of the mucous membrane may be thus induced, keeping up this unpleasant disease a long time, and retarding the restoration of the strength of the patient. Opiates and mild absorbent remedies are the agents upon which we must rely in such cases, and we shall generally find the disease yield to such applications. I have, however, known the irritation transmitted from the ulcerated membrane to the mesenteric glands, and the patient has died, worn out by profuse discharges and hectic fever, after weeks of suffering.

